# Hollow‐Structured Li Hosts Featuring Lithiophilic Metal/Metal Compound Sites for Li‐Metal Anodes

**DOI:** 10.1002/advs.202523923

**Published:** 2026-02-05

**Authors:** Chen Yu, Jiarui Yang, Deyan Luan, Xiong Wen (David) Lou

**Affiliations:** ^1^ Department of Chemistry City University of Hong Kong Kowloon Hong Kong China

**Keywords:** hollow structures, Li‐metal anodes, Li‐metal batteries, lithiophilic hosts, metal compounds

## Abstract

Li‐metal anodes (LMAs) have attracted significant interest in high‐energy‐density Li‐metal batteries (LMBs) due to their high theoretical capacity and low electrochemical potential. Nonetheless, the instability and host‐less nature of Li impede the development of stable LMBs. To address the challenge, metals and metal compounds have been demonstrated to exhibit effective lithiophilicity in guiding uniform Li deposition. Concurrently, hollow‐structured nanomaterials have emerged as advanced Li hosts for LMAs. This review discusses the application of hollow Li hosts featuring metals or metal compounds in LMAs, investigates the in situ and ex situ characterizations of Li deposition behavior in hollow Li hosts, elucidates the mechanisms of regulating Li deposition by metals or metal compounds, and explores the representative instances of hollow Li hosts containing metal or metal compound sites. Finally, this review outlines some future prospects for the advancement of hollow Li hosts integrated with metals or metal compounds.

## Graphical abstract

This review summarizes some major developments of hollow‐structured nanomaterials as advanced Li hosts for Li‐metal anodes (LMAs). In particular, it focuses on hollow Li hosts with metals or metal compounds as the lithiophilic sites. Some future prospects for the advancement of hollow Li hosts are provided.

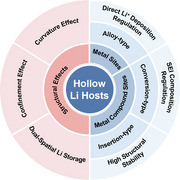

## 1 Introduction

The pressing need for the development of high‐energy‐density energy storage devices is propelled by significant scientific challenges in light of global warming, including the limited range of electric vehicles, the need to mitigate the intermittency of renewable energy sources, and the requirement for long‐term energy storage solutions. Despite the commendable safety index offered by Li‐ion batteries (LIBs), which have been in commercial use for over three decades, their energy density and capacity have been deemed markedly inadequate to satisfy the evolving demands of emerging industries. By utilizing Li‐metal anodes (LMAs) as the anode active material, Li‐metal batteries (LMBs) offer exceptionally high energy density due to the high discharge voltage (−3.04 V versus standard hydrogen electrode) and remarkably theoretical specific capacity (3860 mAh g^−1^) of Li metal [1]. Researchers hold LMAs in high esteem and widely consider them as the “holy grail” of battery research. In 2016, the U.S. Department of Energy launched the Battery500 project to advance LMBs to achieve an energy density as remarkable as 500 Wh kg^−1^ [2, 3]. Nevertheless, LMBs remain a considerable distance on the path to industrialization, hindered by the unstable properties of Li metal. For LMAs, Cu foil is conventionally used as a current collector due to its fitting physical and chemical properties, where Li will plate or strip on the Cu foil (or Cu‐Li)/electrolyte interface throughout each cycling. However, Cu exhibits poor lithiophilicity, resulting in uneven Li plating and the formation of dendrites [4]. Consequently, the unevenly deposited Li metal will be extensively exposed to the electrolyte, triggering undesirable side reactions and unnecessary Li consumption. This will lead to low Coulombic efficiency (CE) and poor cycling performance. Additionally, Li plating on planar Cu foil induces significant volume expansion [5], causing a fragile solid electrolyte interface (SEI) layer to rupture, exposing fresh Li inside, and exacerbating side reactions [6, 7]. Replacing planar Cu foil with 3D macroporous current collectors, such as Cu foam [8, 9, 10], Ni foam [11, 12, 13], etc., can partially alleviate volume expansion. However, the large Li metal surface area exposed to the electrolyte remains as a challenge. Furthermore, this configuration also compromises the volumetric and gravimetric energy densities of LMBs.

Utilizing hollow‐structured nanomaterials as Li hosts has proven to be an effective strategy to address these challenges. These hosts [14, 15], particularly carbon‐based hollow Li hosts, are lightweight, which is favorable to constructing high‐energy‐density LMBs. They typically incorporate embedded lithiophilic sites that reduce the Li nucleation overpotential and induce Li deposition within their internal nanoscale cavities, further effectively segregating the deposited Li from the electrolyte, and inhibiting dendrite formation [16, 17]. Furthermore, the confined Li deposition within the cavities mitigates volume expansion of Li during cycling, promoting the formation of a stable SEI layer [18, 19].

Thanks to the significant development of nanotechnology and electron microscopy techniques, diverse types of hollow Li hosts can be synthesized and characterized at the nanoscale. The synthesis strategies of these hosts are primarily categorized into hard templating methods [20, 21], soft templating methods [22, 23], and self‐templating methods (based on Kirkendall effect or Ostwald ripening, etc.) [24, 25]. Additionally, the introduction of in situ electron microscopy enabled real‐time characterization of Li deposition behavior within hollow hosts [26], offering critical insights for guiding the design and optimization of high‐performance hollow Li hosts.

Certain metals and metal compounds exhibit significant lithiophilicity, promoting uniform Li deposition to achieve stable LMAs, and paving the way toward the industrialization of high‐energy‐density LMBs. The underlying lithiophilic mechanisms are diverse, including alloying reactions [17, 27, 28], conversion reactions [29, 30], and insertion reactions [31, 32]. Coupling metal or metal compound sites with hollow Li hosts has been widely recognized as an effective strategy for constructing high‐performance Li hosts. This review comprehensively summarizes the recent advances in hollow Li hosts featuring metal or metal compound lithiophilic sites for stable LMAs. First, Li deposition behavior in Li hosts with various hollow nano‐structures is systematically summarized, according to the observation by in situ transmission electron microscopy (TEM) and ex situ field emission scanning electron microscopy (FESEM). Subsequently, the mechanisms of metal sites (alloying‐type) and metal compounds (conversion‐ or insertion‐type) in regulating uniform Li plating are discussed by analyzing phase diagrams, density functional theory (DFT) calculations, and in/ex situ X‐ray diffraction (XRD)/selected area electron diffraction (SAED) data. Next, some representative instances of hollow Li hosts with lithiophilic metal or metal compound sites are further highlighted, including mono‐nanoparticle (mono‐NP) hollow Li hosts and hollow nanofibers. Finally, the conclusion and perspectives from hollow Li host design, synthesis, and manufacturing for future development in hollow Li hosts integrated with metals or metal compounds are discussed and emphasized.

## 2 Li Deposition Behavior in Hollow Li Hosts

**FIGURE 1:**
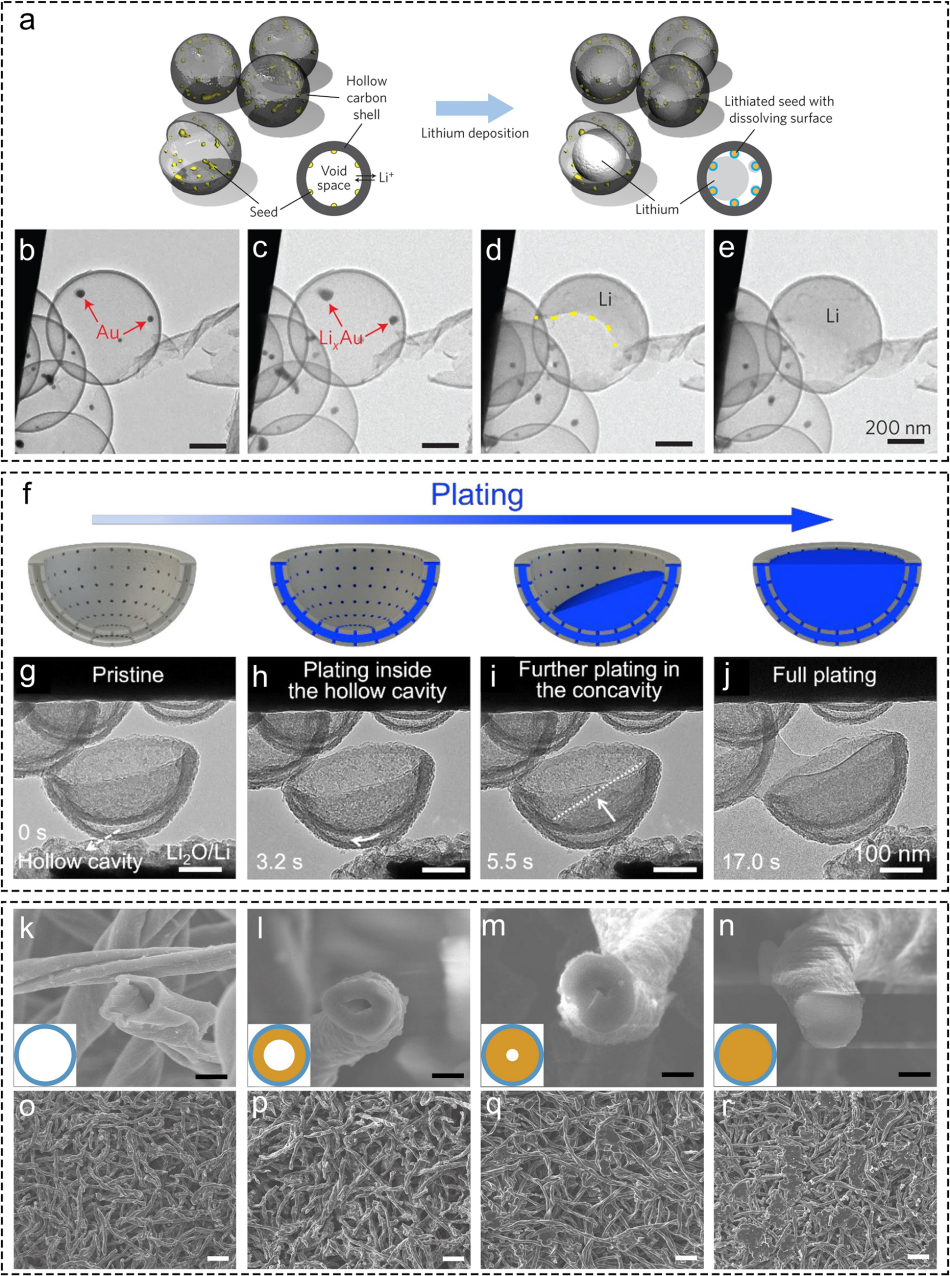
In situ and ex situ characterizations of hollow Li hosts. (a) Schematic illustration of Li deposition within the void space of carbon nanocapsules with Au NPs. (b–e) In situ TEM snapshots of Li plating process in carbon nanocapsules with Au NPs [27]. Copyright 2016, Springer Nature Limited. (f) Schematic illustration of Li deposition behavior in CBs with hollow interlayers. (g–j) In situ TEM snapshots of Li plating process in CBs [43]. Copyright 2021, American Chemical Society. Cross‐sectional FESEM images of 3D HCFs with different Li plating capacities, (k) 0 mAh cm^−2^, (l) 2 mAh cm^−2^, (m) 4 mAh cm^−2^, and (n) 6 mAh cm^−2^. Scale bars, 5 µm. Top‐view FESEM images of 3D HCFs with different Li plating capacities, (o) 0 mAh cm^−2^, (p) 2 mAh cm^−2^, (q) 4 mAh cm^−2^, and (r) 6 mAh cm^−2^. Scale bars, 50 µm [46]. Copyright 2017, Elsevier.

In situ TEM has proven highly effective for characterizing Li deposition behavior within hollow Li hosts. After its initial use in the early 2010s for tracking structural evolution during Li‐ion storage [33, 34, 35], a key breakthrough came in 2014 when Zheng et al. first observed metallic Li deposition behavior on an Au electrode [36]. Later in 2016, Cui et al. employed silica spheres as hard templates to synthesize hollow carbon nanocapsules embedded with Au NPs, which served as a model system for investigating Li deposition behavior in hollow Li hosts [27]. As depicted in Figure 1a, during Li plating process, Li preferentially deposited on the Au NPs, thereby filling the hollow carbon spheres in a liquid‐like manner until their cavities were almost completely filled (Figure 1b–e). This result clearly demonstrates that lithiophilic sites guide preferential deposition of Li, while the hollow nanostructure plays a crucial role in regulating and confining Li deposition. Consistent with this, numerous studies have indicated that Li favors deposition within confined nanoscale spaces [37, 38], such as inside hollow carbon nanotubes [39, 40]. Later in 2020, Ye et al. [38]. observed the Li deposition behavior in nitrogen‐doped hollow porous carbon spheres (N‐HPCSs) by in situ TEM. In the seed‐free hollow carbon spheres, Li initially deposits from one point inside the cavity, subsequently progressing to fill the void space with a relatively flat advancing front surface. Moreover, hollow nanotubes, being a representative 1D hollow nanostructure for their special physicochemical properties such as directional conductivity, their capability of regulating Li deposition has also been investigated. Wang et al. [39] conducted a study on the Li deposition behavior within amorphous carbon nanotubes (aCNTs) by utilizing in situ TEM. For aCNTs with favorable lithiophilicity, the carbon shell will expand initially from Li intercalation. Li subsequently occupies the inner cavity of the nanotubes along the tube walls. Conversely, for aCNTs with limited lithiophilicity, Li will deposit and accumulate on the external surface of the carbon shell. In another investigation [41], for aCNTs embedded with Au NPs, Li predominantly nucleates at the Au NPs and fills the inner space of the carbon nanotubes on both directions. These two studies also highlight the distinct inducing effect of 1D hollow nanostructures on Li deposition. The above discussion illustrates Li deposition behavior within simple confined hollow nanostructures, indicating a clear tendency for Li to deposit within confined nanoscale voids. Previous studies have further suggested that Li preferentially deposits at the interface with the smallest curvature [42]. However, Li deposition behavior in practical LMAs might be more complex. Wang and co‐workers studied the Li deposition sequence on complex hollow carbon nano‐bowls (CBs) by in situ TEM [43]. The CBs, synthesized by a silica‐assisted templating strategy, contain three distinct regions: a semi‐enclosed hemispherical space, a hollow interlayer, and a convex surface (Figure 1f,g). These features make CBs a suitable platform for exploring Li deposition preferences in structurally intricate hosts. Based on in situ TEM observation, the Li deposition sequence in CB is depicted in Figure 1f. Li initially deposits within the hollow structure, then gradually fills the concave space above the nano‐bowl, and eventually accumulates on the convex surface (Figure 1g–j). The sequence suggests a preference for Li deposition in fully enclosed spaces, followed by on the interfaces with negative curvature, and finally on those with positive curvature. In practical scenarios, CBs are densely stacked on the current collector by coating. In this case, Li metal was also observed to first deposit within the hollow interlayers and the gap between adjacent CBs, as these spaces provide a more confined environment. DFT calculation results reveal the preference for Li deposition on confined interfaces with negative curvature from an energy perspective. [43] For N‐doped carbon, the interface with negative curvature exhibits more negative binding energy with Li, which can facilitate Li deposition, while the interface with positive curvature shows more positive binding energy, which hinders Li deposition. This conclusion may also shed light on why Li tends to deposit inside hollow carbon nanocapsules embedded with Au NPs. This study elucidates Li deposition behavior in highly intricate nanostructures, offering valuable insight into the Li deposition dynamics of practical LMAs.

The synthesis of nanofibers has opened new avenues for designing high‐performance Li hosts. These fibers exhibit high specific surface area derived from their unique 3D macroporous structures [44, 45], which helps distribute local current density and inhibit Li dendrite formation. Beyond these advantages, the internal cavities of hollow nanofibers can store a substantial amount of metallic Li, achieving uniform Li deposition [17]. For example, the Guo and Wan group used natural cotton fibers as precursors to produce hollow carbon nanofibers (3D‐HCFs) by a simple annealing process, as shown in Figure 1k. The obtained 3D‐HCFs were employed as a model system and plated with metallic Li with different areal capacities of 0, 2, 4, and 6 mAh cm^−2^ (Figure 1k–r) to investigate the deposition behavior of Li within hollow carbon nanofibers through ex situ FESEM [46]. It can be observed that Li was primarily deposited within the void space of 3D‐HCFs. When the areal capacity increased to 4 mAh cm^−2^, the inner voids of 3D‐HCFs were almost filled. As the areal capacity further reached 6 mAh cm^−2^, metallic Li significantly filled the external macroporous space of 3D‐HCFs. The Li metal deposited in the large pore space remained flat, without any noticeable dendrites observed. This instance showcases the effectiveness of 3D nanofibers in regulating and accommodating Li deposition. Kang et al. [40] utilized in situ TEM to confirm that at relatively low areal capacities, Li deposition is confined inside the hollow porous carbon nanofibers. Hollow nanofibers present a complex hollow Li host system that integrates hollow nanostructures with intertwined and coiled fibers, containing numerous surfaces with positive and negative curvatures. Furthermore, the internal continuous cavities and external 3D mesopores of hollow nanofibers provide a significant volume for Li deposition, which is beneficial for maintaining a low volume change during cycling. This feature prohibits the cracking of SEI layer triggered by substantial volume variations, consequently suppressing side reactions and the formation of dead Li [17, 40, 47].

The research discussed above indicates that Li prefers to deposit within the confined space of hollow nanostructures until these spaces are almost filled with Li. Subsequently, excess Li overflows into the external regions of the hollow structure to continue depositing. This behavior strongly suggests that the internal space of hollow Li hosts can effectively guide Li deposition inward, adequately accommodating Li within their cavities at low areal capacities. The behavior effectively prevents the formation of Li dendrites, mitigates volume expansion of LMAs during cycling, and enhances the stability of SEI layer.

## 3 Mechanism of Lithiophilic Metal/Metal Compound Sites

### 3.1 Mechanism of Lithiophilic Metal Sites: Alloying

**FIGURE 2:**
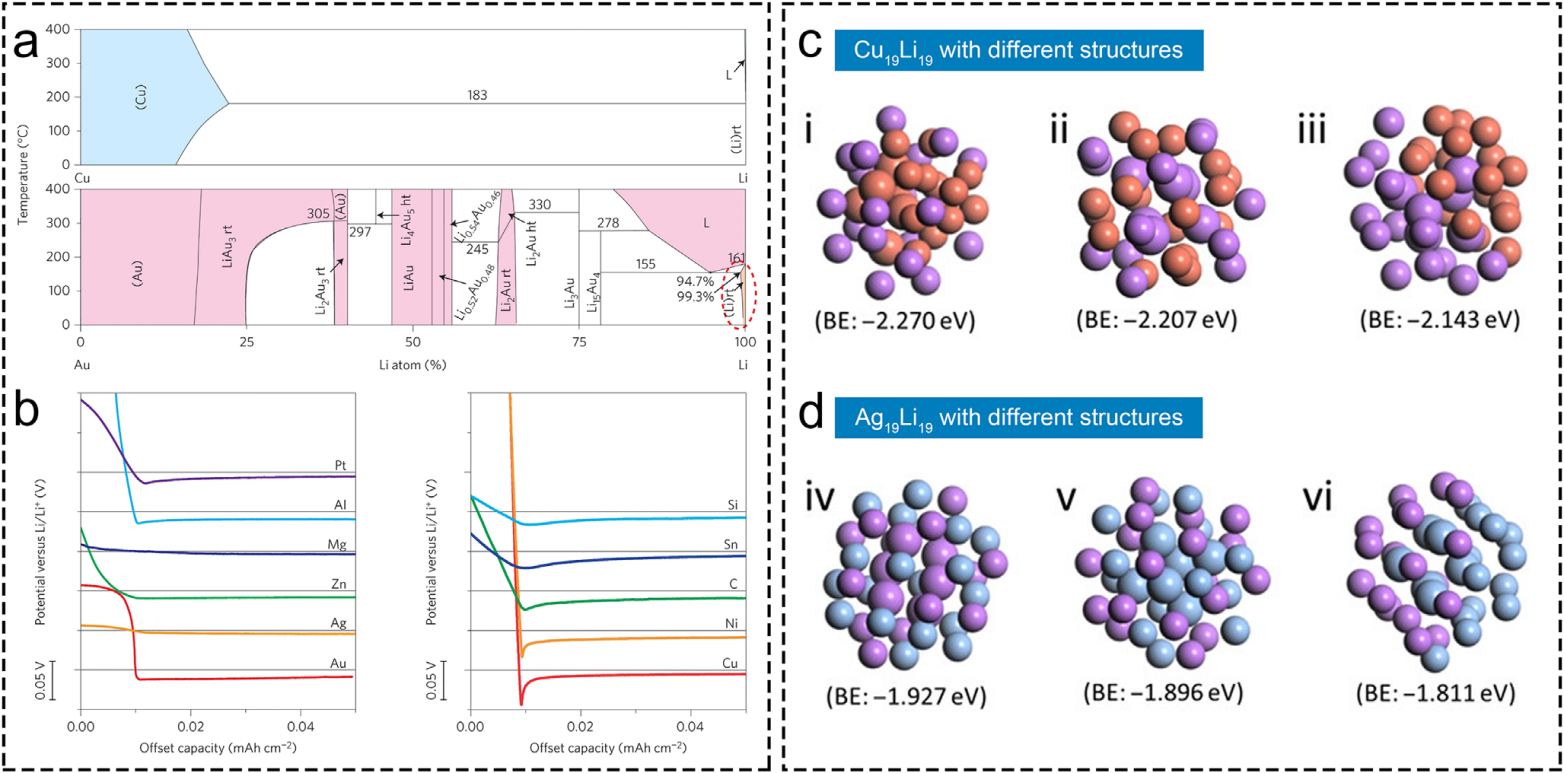
Mechanism of lithiophilic metal sites for regulating uniform Li plating. (a)Li‐Cu (top) and Li‐Au (bottom) phase diagrams. L stands for liquid, and Li (rt) stands for Li metal phase at room temperature. (b) Voltage profiles of Li plating on different substrates with a fixed current density of 10 µA cm^−2^ [27]. Copyright 2016, Springer Nature Limited. Different structures of Cu_19_Li_19_ (c) and Ag_19_Li_19_ (d) and corresponding binding energies (BE). (i) Atom segregation with Cu core, (ii) homogeneous dispersion, (iii) complete atom segregation, (iv) homogeneous dispersion, (v) atom segregation with Ag core, and (vi) complete atom segregation [17]. Copyright 2021, American Association for the Advancement of Science.

Beyond the structural advantages of hollow Li hosts, the introduction of lithiophilic species is equally essential. Uniformly dispersed lithiophilic species are beneficial to induce homogeneous Li deposition, and the underlying mechanisms play a crucial role in determining the electrochemical performance of hollow Li hosts. Among them, highly dispersed lithiophilic metal NPs are investigated early on and primarily induce uniform deposition through alloying reactions. In Figure 2a, the Cu─Li and Au─Li phase diagrams are compared [27]. Cu cannot form an alloy with Li at any atomic ratios, while Au can form various Au*_x_* Li*_y_* alloy phases across a range of compositions. Fang et al. presented the Ag─Li phase diagram, indicating that Ag can form different alloy phases with Li at various atomic ratios [17].

Additionally, there are significant differences in the nucleation overpotential (versus Li^+^/Li) of Li on different substrates. Theoretically, Li nucleation commences when the potential reaches to or below 0 V (versus Li^+^/Li). However, in practice, concentration polarization often requires Li^+^ to overcome a certain overpotential to drive the nucleation process [48]. A lower absolute value of nucleation overpotential suggests easier nucleation on the substrate [49]. Therefore, at a fixed current density, the nucleation overpotential is commonly used to quantitatively evaluate the lithiophilicity of the substrate [50]. As shown in Figure 2b, Cu, which does not form alloys with Li, exhibits a large Li nucleation overpotential during Li plating [27]. In contrast, Au and Ag, which undergo alloying, show considerably lower overpotentials. Figure 2b also illustrates Li nucleation overpotentials on a range of metal substrates. Apart from Au and Ag, ultra‐low Li nucleation overpotentials are also realized on Mg, Zn, etc., indicating significant lithiophilicity of these metals. Many studies have reported that Li can easily form alloys with Mg [29, 51, 52], Zn [53], and other metals such as Sn [54, 55, 56] and In [53, 57, 58]. The ease with which a specific metal forms an alloy with Li can also be assessed by comparing the concentration range over which the metal can form an alloy with Li and the diversity of alloy types, as depicted in the binary phase diagram of the metal with Li. The nucleation overpotential on a substrate reflects the characteristics of the Li‐substrate phase diagram from an electrochemical perspective. When designing Li hosts, it is advisable to select preferentially metals such as Zn, Sn, etc. as functional sites that are easily alloyed with Li (exhibit high lithiophilicity), cost‐effective, and can be easily prepared.

DFT calculations provide a more in‐depth theoretical understanding of the alloying reactions between metal sites and Li, and their impact on Li deposition uniformity. Fang et al. used Cu_19_Li_19_ and Ag_19_Li_19_ as model systems to construct three different cluster models, including (i) atom segregation with a Cu core, (ii) homogeneous dispersion, (iii) complete atom segregation, (iv) homogeneous dispersion, and (v) atom segregation with an Ag core [17]. The binding energies of these models were calculated (Figure 2c,d). The results indicate that Cu_19_Li_19_ species tend to exhibit atom segregation, where Cu and Li are not evenly dispersed. While Ag_19_Li_19_ species tend to uniformize atomic dispersion, with Ag and Li merging homogeneously. The results support the macroscopic phenomenon that lithiophobic Cu hardly alloys with Li, while lithiophilic Ag readily forms alloys. The DFT results provide an atomic‐level explanation of some information in Li─Cu and Li─Ag phase diagrams.

The lithiophilicity of metal sites in Li hosts is generally achieved through relatively straightforward alloying reactions. In contrast, the composition and properties of the SEI layer are critical for determining the cycling performance of batteries. During battery cycling, temperature plays a significant role in SEI composition for LMBs [59, 60]. However, the composition of the Li host and the electrolyte typically has a more pronounced impact on SEI composition. The SEI composition is primarily influenced by the decomposition of anions in the electrolyte or reactions between the anions in Li host and Li^+^. SEI formation usually initiates with the development of tiny nuclei at the anode/electrolyte interface, which then gradually expands to cover the entire interface. The internal structure of SEI is often believed as mosaic‐like [61, 62, 63], and its composition can typically be deduced from X‐ray photoelectron spectroscopy (XPS) results [64, 65]. A robust and potent SEI layer enables the efficient transport of Li^+^ ions. During the Li plating process, Li^+^ ions move through the SEI layer and deposit between the SEI and the anode, while during Li stripping, Li^+^ ions pass through the SEI layer and dissolve into the electrolyte. The SEI layer consistently acts as a protective screen for LMAs during battery cycling. Therefore, it is vital to construct a well‐formed SEI layer with beneficial components that meet the requirements of lithiophilicity, electrochemical properties, and structural integrity of LMAs.

Components such as LiF and Li_2_O within the SEI layer provide favorable mechanical stability and low electronic conductivity, which are crucial for maintaining structural integrity and impeding electronic conduction, thereby preventing battery short circuits [66, 67, 68, 69]. However, recent reports have raised doubts regarding the unequivocal benefits of LiF [70, 71]. This highlights the potential of Li hosts that can promote the formation of beneficial SEI components to improve the cycling performance of LMAs. However, direct regulation of the SEI component by lithiophilic metal sites has received limited attention.

### 3.2 Mechanism of Lithiophilic Metal Compound Sites: Conversion

Numerous metal compound sites undergo conversion reactions with Li^+^ during charging, promoting the formation of a desirable SEI composition. The general reaction principle is as follows:

$$Li^{+}+e^{-}+M-X\to \mathrm{Li}-M+\mathrm{Li}-X$$

where M stands for metal elements, X stands for non‐metal elements or anionic groups.

**FIGURE 3:**
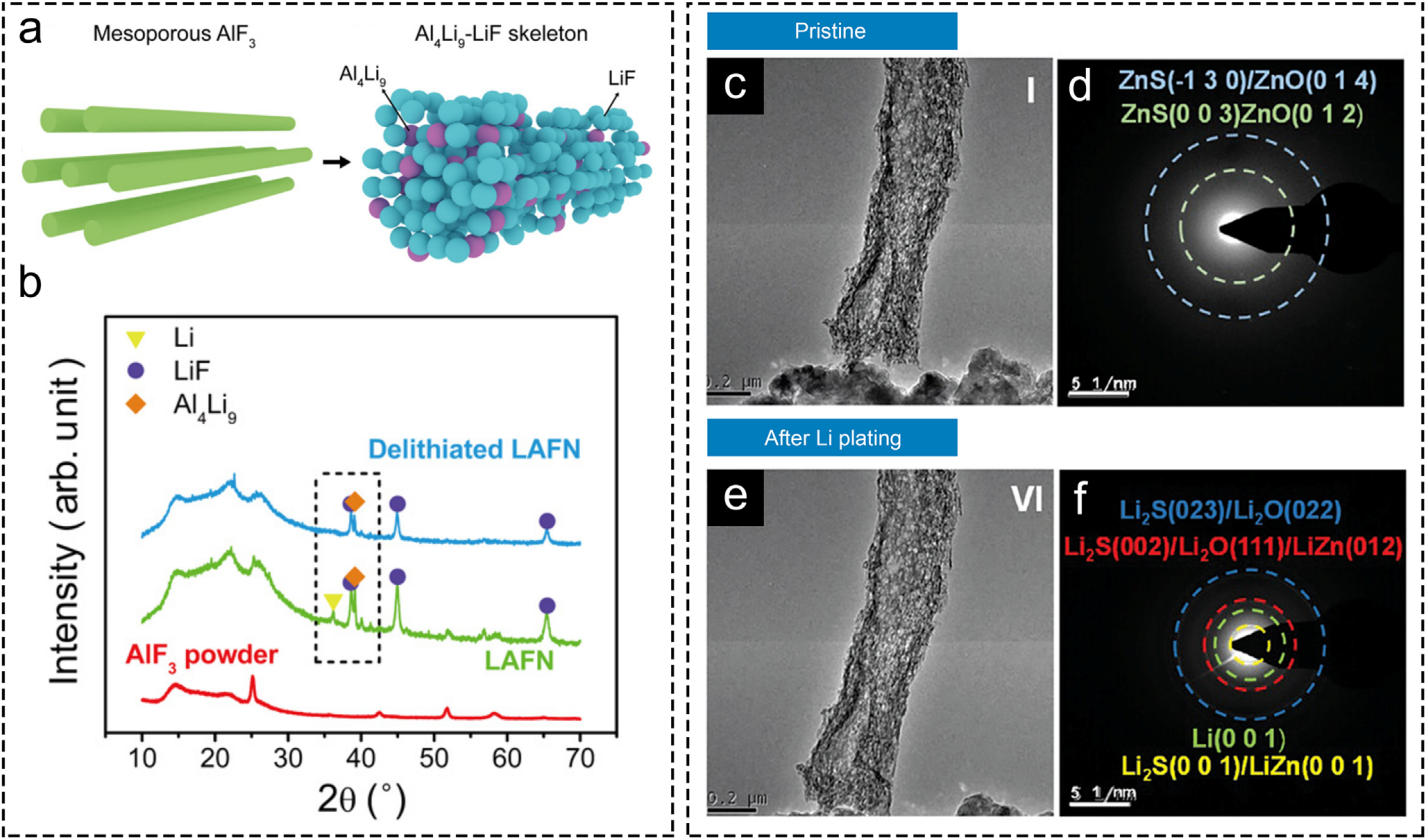
Mechanism of regulating Li plating by conversion‐type metal compounds. (a) Schematic illustration of the conversion reaction between mesoporous AlF_3_ and Li. (b) XRD patterns of LAFN electrode before and after stripping Li to 1 V (versus Li^+^/Li) [72]. Copyright 2017, American Association for the Advancement of Science. In situ TEM snapshots and corresponding SAED patterns of ZOS‐CF (c,d) before and (e,f) after Li plating process [30]. Copyright 2025, Wiley‐VCH.

The reduction of a metal compound can yield both metal sites and Li compounds. If the produced Li compound is a favorable SEI component, this conversion reaction can improve the cycling performance of LMAs. Based on this principle, Cui's group conducted a reaction between mesoporous AlF_3_ and metallic Li, obtaining LAFN consisting of Al_4_Li_9_‐LiF with excess Li (Figure 3a,b) [72]. XRD analysis confirmed that both Al_4_Li_9_ and LiF components in LAFN were well‐retained upon delithiation, serving as lithiophilic sites and favorable SEI components (Figure 3b). Despite the Al─Li alloy and LiF in this instance were not formed during cycling, this example highlights the noteworthy reactivity between Li and specific metal compounds, leading to the formation of lithiophilic sites and favorable SEI components. Wang and co‐workers synthesized Bi_2_O_3_‐modified Cu foam (BO@CF) as a Li host. The ex situ XRD pattern and XPS of lithiated BO@CF revealed clear peaks of Li_3_Bi and enhanced Li_2_O signals, respectively, confirming the conversion reaction of Bi_2_O_3_ with Li during electrochemical lithiation [73]. The reaction between metal compounds and Li can also be directly observed through the evolution of the SAED pattern by in situ TEM observation during Li plating process. Figure 3c,d show in situ TEM snapshots of carbon fibers decorated with ZnS and ZnO NPs, along with the corresponding SAED patterns [30]. The SAED patterns of the pristine sample contain diffraction rings related to ZnS and ZnO. Upon lithiation, these original diffraction rings disappeared and were replaced by new diffraction rings related to Li, LiZn alloy, Li_2_S, and Li_2_O (Figure 3d,f). The result suggests the occurrence of conversion reactions on the sample, resulting in the formation of LiZn, Li_2_S, and Li_2_O. Furthermore, other metal compounds such as metal fluorides, phosphides, and nitrides can undergo similar conversion reactions during the lithiation process, generating of SEI‐favorable LiF [74, 75, 76], Li_3_P [77], and Li_3_N [78].

### 3.3 Mechanism of Lithiophilic Metal Compound Sites: Insertion

**FIGURE 4:**
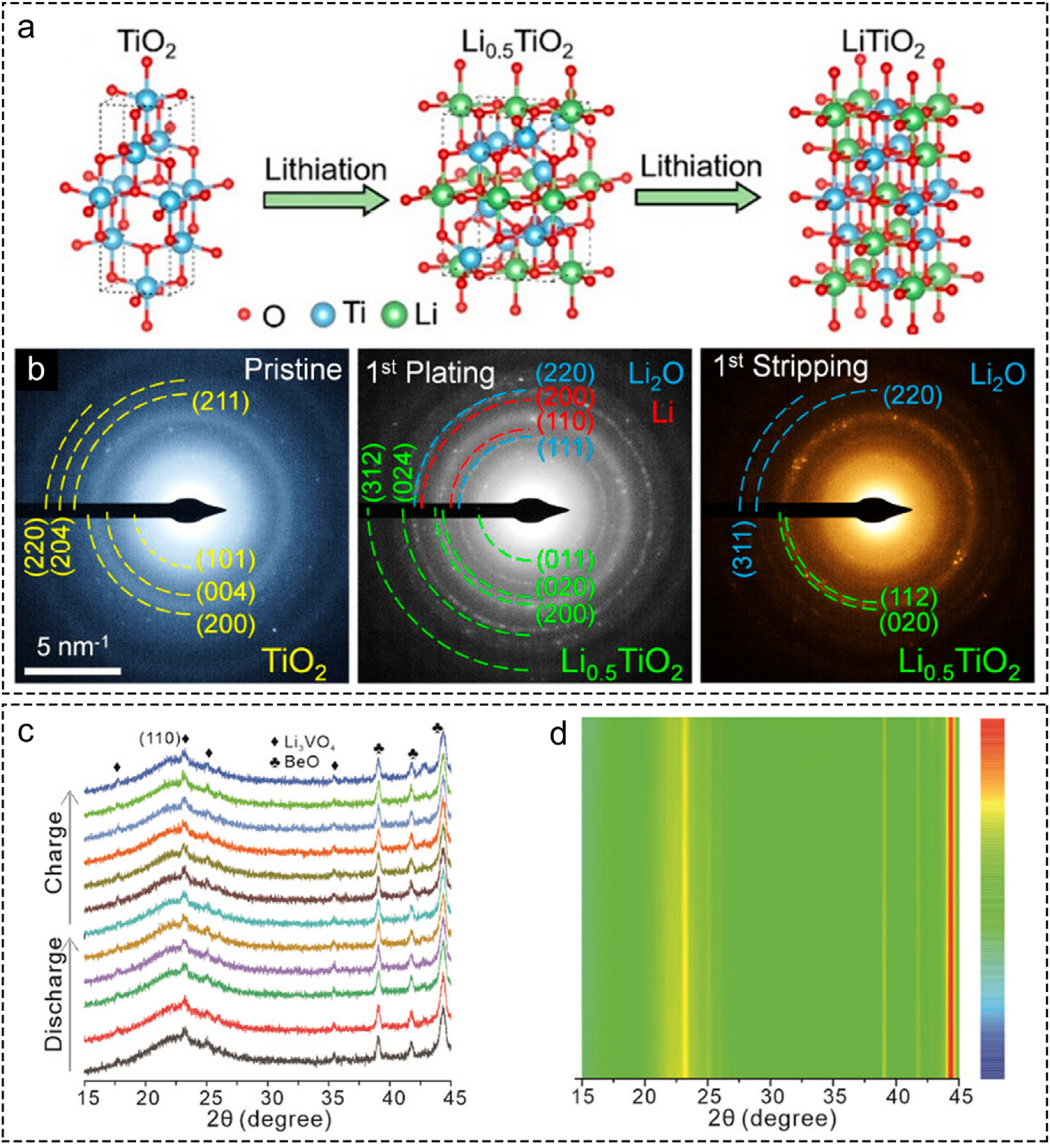
Mechanism of regulating Li plating by insertion‐type metal compounds. (a) Schematic illustration of Li insertion process in TiO_2_. (b) SAED patterns of TiO_2_@N‐HPCSs before and after first plating and first stripping [31]. Copyright 2022, American Chemical Society. In situ XRD patterns (c) and corresponding contour plots (d) of LVO@CNFs anode during the initial cycle [32]. Copyright 2025, Wiley‐VCH.

Most metal compounds exhibit lithiophilicity through conversion reactions. A distinct category, however, exhibits lithiophilicity through insertion reactions with Li^+^ due to its special crystal structure, with TiO_2_ being a prime example. During the initial stages of lithiation, Li and TiO_2_ will form a Li_x_TiO_2_ (where x < 0.5) solid solution. Continued Li^+^ insertion generates Li_0.5_TiO_2_, which subsequently transits to LiTiO_2_ upon further lithiation (Figure 4a). This mechanism is verified through the evolution of SAED patterns during TiO_2_ lithiation (Figure 4b). The resulting LiTiO_2_ is lithiophilic and facilitates uniform Li deposition. Notably, the insertion mechanism induces the minimal increase in lattice spacing and volume expansion in TiO_2_ [31]. Therefore, employing TiO_2_ as a Li host can effectively alleviate the volume expansion resulting from Li deposition [31].

Beyond TiO_2_, Li_3_VO_4_ also undergoes Li insertion reactions. Sun and co‐workers fabricated carbon nanofibers decorated with Li_3_VO_4_ nanodots (LVO@CNFs) as a Li host and investigated its insertion behavior [32]. Figure 4c,d presents the in situ XRD patterns and corresponding contour plots during the first cycle of LVO@CNFs in a half cell. The negligible changes of diffraction peaks of Li_3_VO_4_ indicate the almost absence of phase transformation during Li plating, confirming an insertion mechanism between Li^+^ and Li_3_VO_4_. Nevertheless, the design of hollow Li hosts utilizing Li_3_VO_4_ as an insertion‐type lithiophilic site has rarely been reported.

## 4 Hollow Li Hosts Featuring Lithiophilic Metal/Metal Compound Sites

### 4.1 Hollow Li Hosts Featuring Alloy‐type Metal Sites

**FIGURE 5:**
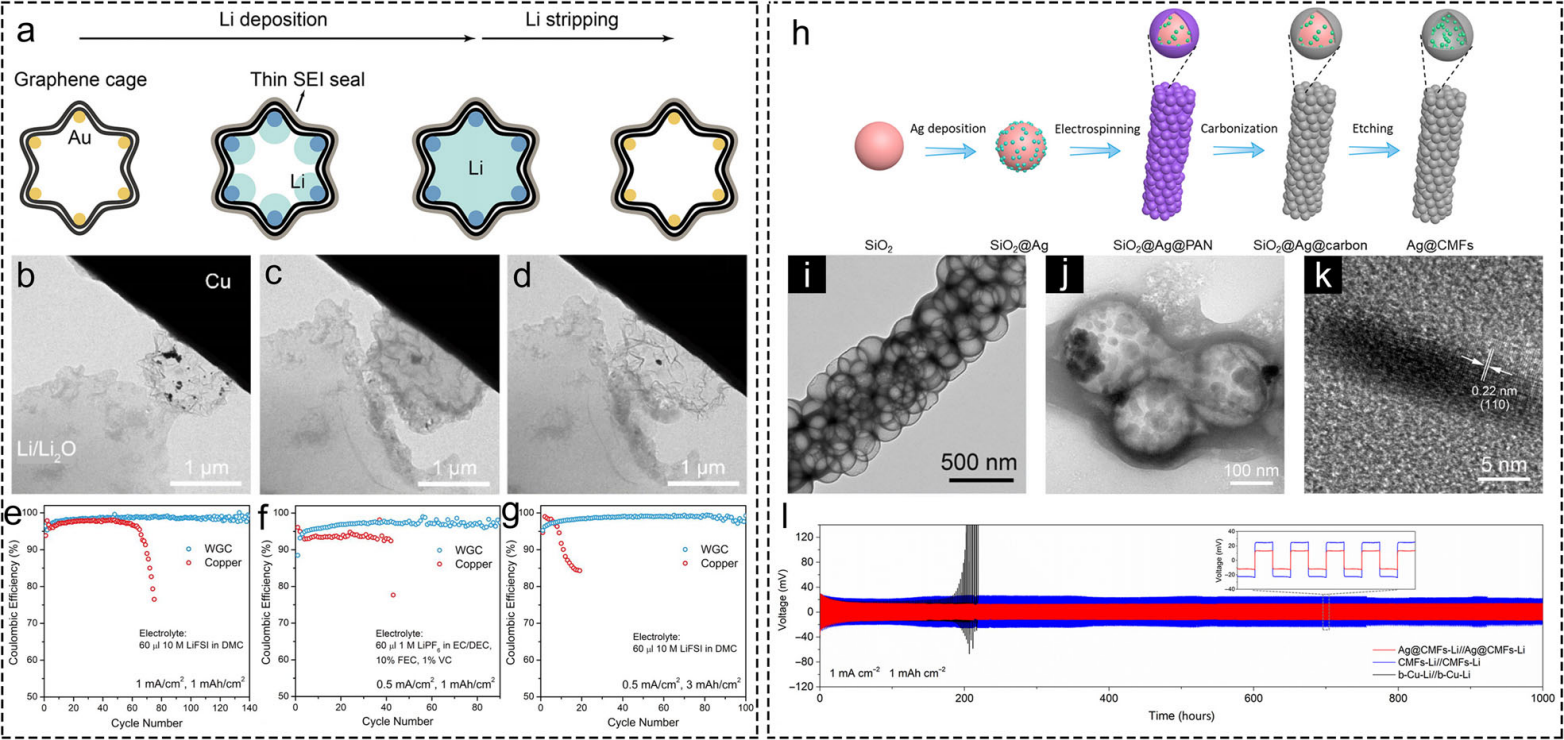
Hollow Li hosts featuring lithiophilic noble metal sites. (a) Schematic illustration of Li deposition and stripping process in WGC. In situ TEM snapshots of (b) pristine, (c) Li plated, and (d) Li stripped status. CE comparison between WGC and bare copper electrodes at (e) 1 mA cm^−2^ and 1 mAh cm^−2^ (f) 0.5 mA cm^−2^ and 1 mAh cm^−2^ and (g) 0.5 mA cm^−2^and 3 mAh cm^−2^ in different electrolytes [28]. Copyright 2019, American Chemical Society. (h) Synthesis approach of Ag@CMFs. (i) TEM images of Ag@CMFs. TEM (j) and (k) HRTEM images of Ag@CMFs after being plated with 2 mAh cm^−2^ Li. (l) Cycling stability comparison of Ag@CMFs‐Li, CMFs‐Li and b‐Cu‐Li electrodes in symmetric cells at 1 mA cm^−2^ and 1 mAh cm^−2^ [17]. Copyright 2021, American Association for the Advancement of Science.

Since the inception of research on Li hosts, lithiophilic metals are among the materials that are studied in the early times for regulating Li deposition. For instance, the Cui group decorated Au NPs within wrinkled graphene cages (WGC) [28]. Figure 5a illustrates the schematic of Li plating and stripping process of WGC. Owing to the presence of lithiophilic Au NPs within WGC, Li^+^ will initially deposit on the Au NPs to form an Au─Li alloy, accompanied by the formation of SEI layer. With continued deposition, the internal cavity of the WGC was gradually filled with Li metal. Upon Li stripping, the hollow structure of WGC was reverted (Figure 5b–d). Half cells constructed with WGC and Li metal demonstrated notably enhanced CE and stability compared to Cu current collectors at various current densities and areal capacities in different electrolytes (Figure 5e–g). Nonetheless, the scarcity and high cost of Au limit its practical application in LMAs, underscoring the need to develop hollow Li hosts based on high‐natural‐abundance and cost‐effective lithiophilic materials.

Furthermore, according to Sand's equation, excessively high local current density will lead to the formation of Li dendrites in LMAs [49, 79]. In view of this, designing Li hosts with high specific surface areas is a potent strategy to effectively disperse local current density and suppress dendrite formation. Nanofibers are considered as promising candidates for Li hosts due to their exceptionally high specific surface area. Besides, the low density of nanofiber Li hosts confers benefits for constructing high‐energy‐density LMBs. Based on the discussion above, the macroporous property of nanofibers also offers volume for metallic Li accommodation. Moreover, electrospinning technology offers an opportunity to enhance the customizability of the nanofiber in nanostructure and chemical composition [80]. By utilizing electrospinning techniques, nanofibers can be engineered to incorporate hollow nanostructures such as hollow nanospheres [81, 82, 83], nanoboxes [44, 84], and hollow nanoprisms [19]. Additionally, hard templating methods can be employed to construct single‐channel [85, 86], multi‐channel [87, 88], and necklace‐like [16, 89] hollow nanofibers, expanding the design possibilities of Li hosts.

Capitalizing on these advantages, Lou's group integrated hollow carbon spheres embedded with lithiophilic Ag NPs into carbon nanofibers (Ag@CMFs) (Figure 5h) [17]. The hollow Ag@CMFs offer both inner void space and outer macroporous space for accommodating deposited Li and alleviating volume change. Additionally, the high specific surface area of Ag@CMFs can significantly reduce local current density, thereby inhibiting dendrite growth. TEM images of Ag@CMFs before and after Li plating (2 mAh cm^−2^)) reveal that Li was predominantly deposited in the interior space of hollow nanospheres, demonstrating the remarkable Li deposition guiding ability of Ag NPs and confined void space (Figure 5i,j). High‐resolution TEM (HRTEM) confirmed the formation of an Ag‐Li alloy after plating (Figure 5k), indicating that the Ag NPs in Ag@CMFs effectively promote Li deposition within the hollow nanospheres by forming Ag─Li alloy. In a symmetric cell, the Ag@CMFs‐Li electrodes demonstrated significantly longer cycling life and lower voltage polarization than both bare Cu (b‐Cu)─Li and CMFs‐Li electrodes, highlighting the beneficial role of Ag NPs in facilitating Li plating (Figure 5l). This work has earlier designed lithiophilic metal‐modified hollow carbon nanofibers and demonstrated its feasibility as highly stable Li hosts.

**FIGURE 6:**
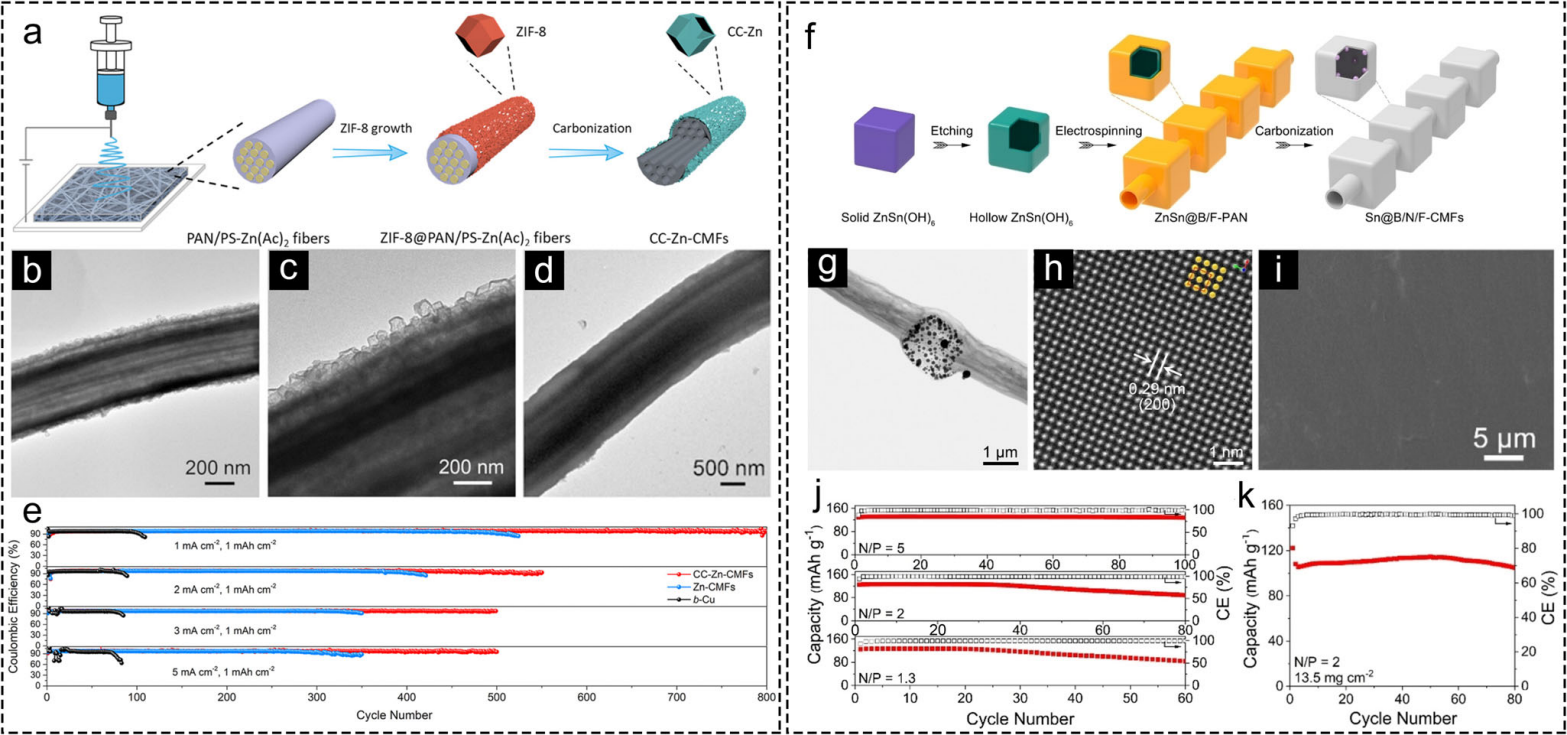
Hollow Li hosts featuring lithiophilic transition metal sites. (a) Synthesis approach of CC‐Zn‐CMFs. TEM images of CC‐Zn‐CMFs (b,c) before and (d) after plated with 2 mAh cm^−2^ Li. (e) CE comparison between CC‐Zn‐CMFs, Zn‐CMFs, and b‐Cu electrodes [90]. Copyright 2021, Wiley‐VCH. (f) Synthesis approach of Sn@B/N/F‐CMFs. (g) Typical and (h) atomic‐resolution HAADF‐STEM images of Sn@B/N/F‐CMFs. (i) Top‐view FESEM image of Sn@B/N/F‐CMFs after plated with 40 mAh cm^−2^ Li. Cycling performance of Sn@B/N/F‐CMFs‐Li//5V‐LNMO full cells under (j) different N/P capacity ratios and (k) N/P capacity ratio of 2 with a cathode loading of 13.5 mg cm^−2^ [16]. Copyright 2025, Wiley‐VCH.

While Ag is more cost‐effective than Au, its expense remains non‐negligible. Leveraging low‐cost transition metals offers a more economically viable pathway for designing hollow Li hosts. Figure 6a illustrates the fabrication of nanofibers utilizing polystyrene (PS) as a sacrificial template and modification by zeolitic imidazolate framework‐8 (ZIF‐8) NPs, resulting in multi‐channel lotus‐root‐like carbon microfibers (CC‐Zn‐CMFs) after carbonization. The resulting CC‐Zn‐CMFs feature hollow carbon nanocages decorated with cost‐effective Zn metal on the outer surface [90]. A comparison of TEM images before and after Li plating (2 mAh cm^−2^) reveals that the multi‐channels effectively confine Li deposition within the internal voids, avoiding excessive Li plating on the exterior of nanofibers and dendrite formation (Figure 6b–d). Half cells assembled with free‐standing CC‐Zn‐CMFs electrodes exhibited stable cycling with an impressive CE compared with b‐Cu electrodes at various current densities (Figure 6e). Furthermore, the hollow carbon nanocages on the surface of CC‐Zn‐CMFs contributed to mitigating volume changes during cycling. This can be demonstrated by comparing the CE stability of CC‐Zn‐CMFs with Zn‐CMFs (Figure 6e).

Another work by Lou and coworkers explored the function of hollow nanofibers in accommodating high‐capacity Li metal. In this research, necklace‐like B, N, F co‐doped carbon nanofibers embedded with Sn NPs (Sn@B/N/F‐CMFs) were synthesized (Figure 6f) [16]. Metallic Sn has been widely proven to possess outstanding lithiophilicity [91, 92, 93], while the co‐doping of B, N, and F further enhances the lithiophilicity of carbon nanofibers. Typical high‐angle annular dark‐field scanning transmission electron microscope (HAADF‐STEM) image of Sn@B/N/F‐CMFs indicates that the Sn NPs are predominantly concentrated within hollow nanoboxes (Figure 6g), with an atomic‐resolution HAADF‐STEM image revealing Sn exposing the (200) plane (Figure 6h). The Sn@B/N/F‐CMFs exhibited flat Li plating without obvious dendrites even at a high areal capacity of 40 mAh cm^−2^, demonstrating their feasibility for high‐capacity LMAs (Figure 6i). Furthermore, the Sn@B/N/F‐CMFs‐Li exhibit an excellent compatibility with LiNi_0.5_Mn_1.5_O_4_ (LNMO) cathode in 5V‐class anode‐less LMBs under low negative/positive (N/P) capacity ratios and high cathode loading (Figure 6j,k). This work demonstrates a host material that meets the requirements for both high‐capacity LMAs and high‐energy‐density LMBs.

In addition, introducing multiple different metals into hollow Li hosts and tuning the ratio of these metals represents a strategy for achieving stable LMAs. For instance, Chen et al. synthesized carbon nanofibers composed of hollow nano‐prisms derived from Ni‐Co bimetallic materials through simple electro‐spinning and annealing processes [19]. The nanofibers exhibited an internal lotus root‐like structure. The study revealed that adjusting the amount of metal salts could affect the size of the hollow nano‐prism structures, and influencing the electrochemical and structural stability of the synthesized nanofibers.

Many studies have successfully controlled Li deposition by leveraging the nano‐confinement effect of hollow nanostructures in conjunction with the alloying reactions of metal sites with Li, leading to excellent stability of LMAs. Research in this field has evolved from the utilization of lithiophilic noble metal sites to more cost‐effective transition metal sites as alternatives, thereby enhancing the economic viability of Li host synthesis while improving LMA stability. However, as previously discussed, lithiophilic metal sites lack control over SEI composition. Consequently, there has been a significant focus on the vigorous development of hollow Li hosts featuring conversion‐type metal compound sites.

### 4.2 Hollow Li Hosts Featuring Conversion‐type Metal Compound Sites

**FIGURE 7:**
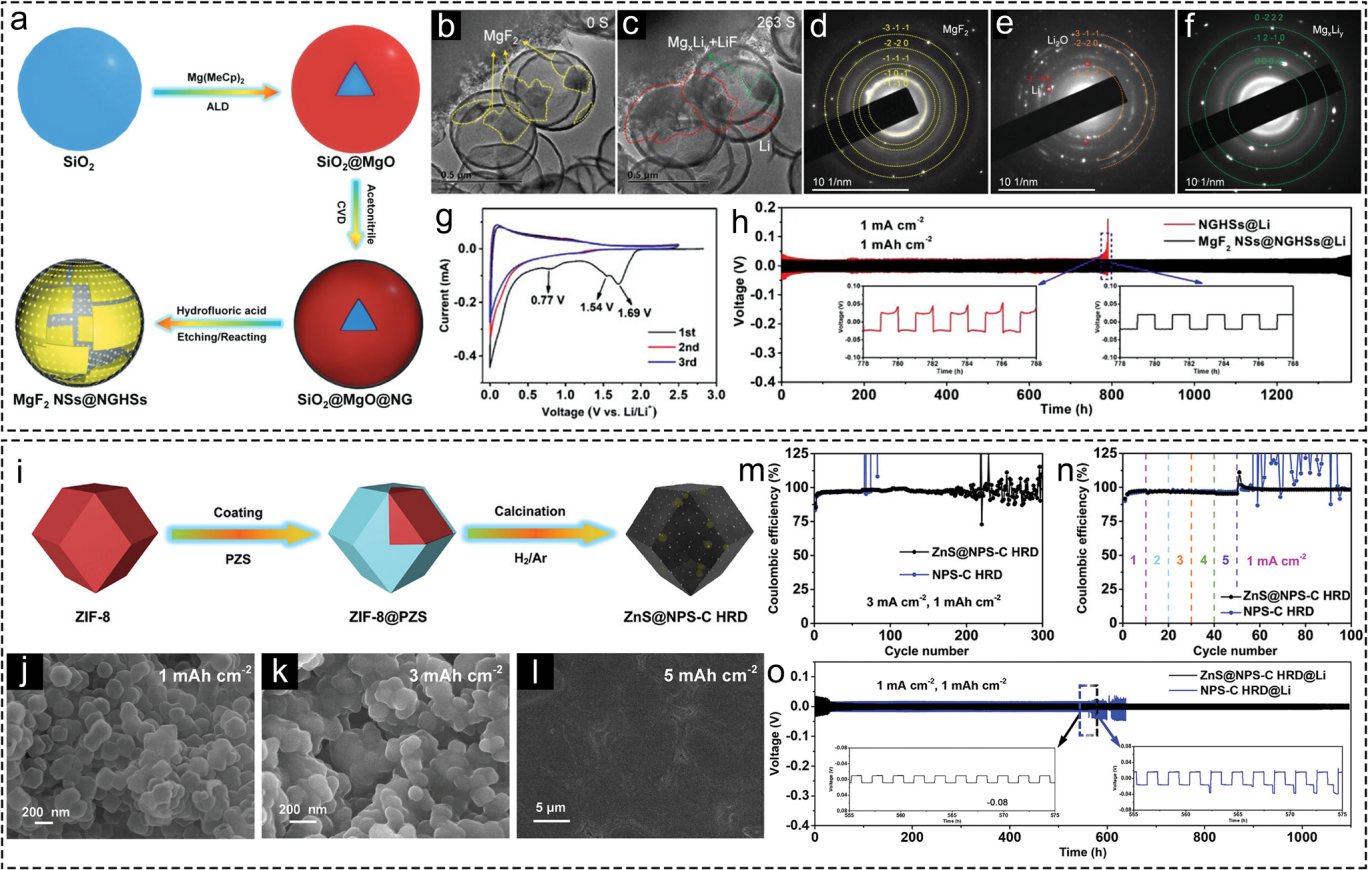
Hollow Li hosts featuring lithiophilic convertion‐type metal compound sites. (a) Schematic illustration of the synthesis of MgF_2_ NSs@NGHSs. (b,c) In situ TEM snapshots of MgF_2_ NSs@NGHSs during Li plating process. SAED patterns of (d) pristine, (e) lithiated, and (f) de‐lithiated MgF_2_ NSs@NGHSs. (g) CV profile of MgF_2_ NSs@NGHSs//Li half cell during the initial 3 cycles. (h) Comparison of cycling stability of NGHSs@Li and MgF_2_ NSs@NGHSs electrodes in symmetric cells at 1 mA cm^−2^ and 1 mAh cm^−2^ [29]. Copyright 2022, Wiley‐VCH. (i) Synthesis approach of ZnS@NPS‐C HRD. Top‐view FESEM images of ZnS@NPS‐C HRD electrode after plated with (j) 1 mAh cm^−2^, (k) 3 mAh cm^−2,^ and (l) 5 mAh cm^−2^ Li. CE comparison of ZnS@NPS‐C HRD and NPS‐C HRD electrodes at (m) constant and (n) mutative current densities. (o) Comparison of cycling stability of ZnS@NPS‐C HRD@Li and NPS‐C HRD@Li electrodes in symmetric cells [97]. Copyright 2024, Wiley‐VCH.

As mentioned earlier, a notable category of metal compound sites undergoes conversion reactions with Li to achieve stable LMAs. For example, Li et al. utilized SiO_2_ as a spherical template to fabricate hollow carbon spheres incorporating MgF_2_ nanosheets (MgF_2_ NSs@NGHSs) (Figure 7a,b,d) [29]. In situ TEM observation revealed that Li preferentially deposits on the MgF_2_ nanosheets (Figure 7b,c). Besides, the conversion reaction between MgF_2_ and Li (MgF_2_ + 2Li = 2LiF + Mg) was verified by the evolution of SAED patterns during lithiation/delithiation (Figure 7d–f), as well as by a distinct peak at 1.54 V in the cyclic voltammetry (CV) curve of the initial cycle of the half cell (Figure 7g). Symmetric cell with MgF_2_ NSs@NGHSs electrodes achieved impressive cycling stability for over 1300 h at 1 mA cm^−2^ and 1 mAh cm^−2^ (Figure 7h). As discussed above, the MgF_2_ nanosheets impart remarkable electrochemical performance to the hollow carbon spheres. Nevertheless, this work introduced hazardous hydrofluoric acid in the preparation of MgF_2_. Exploring safer fluorination methods is essential for synthesizing metal fluoride as conversion‐type lithiophilic sites.

In view of this, Wang et al. synthesized lithiophilic CoF_2_@C hollow spheres (CoF_2_@C‐HS) as Li hosts to regulate Li plating and form a LiF‐rich SEI layer [94]. Compared to the synthesis of MgF_2_ NSs@NGHSs, the fluorination process of Co in this work was achieved by mixing the precursor with polytetrafluoroethylene (PTFE) and annealing the mixture under an inert atmosphere, ensuring a safer fluorination process. The CoF_2_@C/Cu electrode exhibited outstanding electrochemical stability in both half cells and symmetric cells. Upon discharging the CoF_2_@C‐HS//Li cell to 0 V, the HRTEM images of CoF_2_@C‐HS‐Li showcase a transformation in lattice fringes from 0.179, 0.261, and 0.334 nm, corresponding to the (211), (101), and (110) crystal planes of CoF_2_, to 0.204 nm for the (111) plane of Co and 0.200 nm for the (200) plane of LiF. These dynamic changes in lattice fringes validate the conversion mechanism between CoF_2_ and Li. XPS spectra of the cycled CoF_2_@C/Cu electrode suggest the formation of a LiF‐rich SEI layer from the reaction of CoF_2_ and Li, which enhances mechanical and electrochemical stability, facilitates Li^+^ diffusion, and promotes uniform and dense Li deposition [95, 96].

In a work by Liu and co‐workers, ZnS NPs were encapsulated in N, P, S co‐doped hollow rhombic dodecahedral carbon shells (ZnS@NPS‐C HRD) by wrapping poly(cyclotriphosphazene‐co‐4,4′‐sulfonyldiphenol) (PZS) layer on the surface of ZIF‐8 rhombic dodecahedra, followed by a carbonization process (Figure 7i) [97]. The N, P, S co‐doping enhances the lithiophilicity of the carbon shell. During Li plating process, ZnS undergoes a conversion reaction, forming a LiZn alloy and Li_2_S, which was confirmed by CV curves of the half cell. The ZnS@NPS‐C HRD electrode exhibited dendrite‐free and flat Li deposition even at 5 mAh cm^−2^ (Figure 7j–l). The unique hollow nanostructure and conversion reaction between Li and ZnS@NPS‐C HRD contribute to its stable electrochemical performance. The ZnS@NPS‐C HRD//Li half cell demonstrated stable CE values at 3 mA cm^−2^ and 1 mAh cm^−2^ (Figure 7m), and excellent rate performance (Figure 7n). Additionally, the ZnS sites also play a great role in reducing voltage polarization and extending cycling life of symmetric cell (Figure 7o). While the work attributed the performance to the hollow structure and conversion reaction, it did not fully emphasize the role of Li_2_S in stabilizing the LMAs. There are many reports that have confirmed that when integrated as a component of SEI layer, Li_2_S can offer high ionic conductivity to suppress the formation of Li dendrites [98, 99, 100, 101]. Compared to lithiophilic metal sites featured on hollow Li hosts, conversion‐type metal compounds enable direct control over the SEI composition in LMAs. Additionally, the diversity of conversion‐type metal compounds presents great opportunities for stabilizing LMAs.

### 4.3 Hollow Li Hosts Featuring Insertion‐type Metal Compound Sites

**FIGURE 8:**
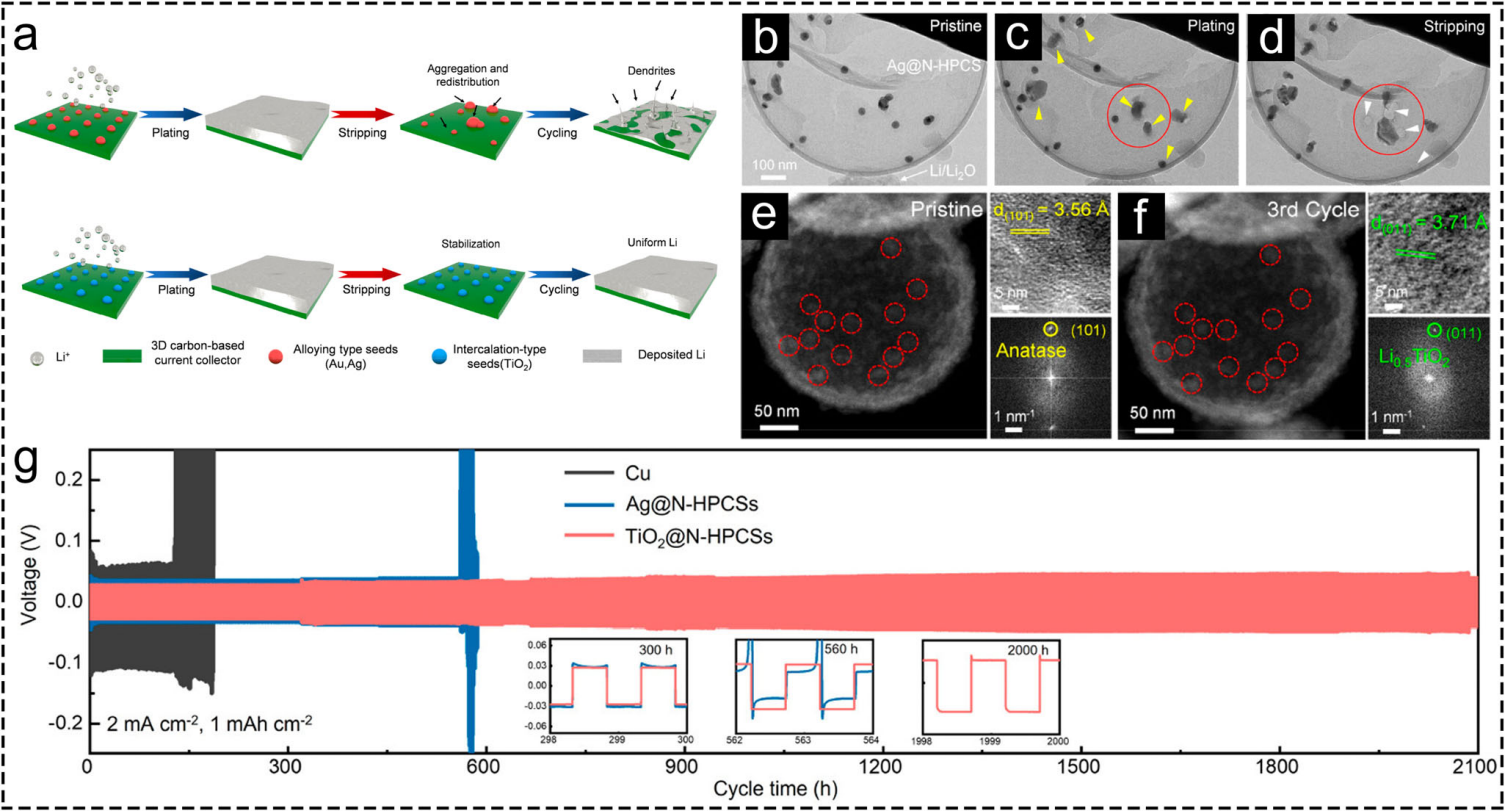
Hollow Li host featuring lithiophilic insertion‐type metal compound sites. (a) Schematic diagram of comparing the uniformity of Li plating on carbon‐based substrates with alloy‐type sites and insertion‐type sites (TiO_2_). In situ TEM snapshots of Ag@N‐HPCs before (b) and after Li plating (c) and stripping (d) process. HAADF‐STEM, HRTEM images and FFT patterns of TiO_2_@N‐HPCSs before (e) and after 3 cycles (f). (g) Cycling stability of Cu, Ag@N‐HPCs and TiO_2_@N‐HPCs electrodes with a certain amount of Li preloaded in symmetric cells [31]. Copyright 2022, American Chemical Society.

The alloying reactions between metal sites and Li often lead to significant volume expansion during Li plating. During repeated cycling, these metal sites tend to aggregate, gradually resulting in an uneven distribution of lithophilic sites that undermines long‐term uniform Li deposition.^31^ Recent reports suggested that even conversion‐type lithiophilic sites will aggregate during cycling, which is not conducive to long‐term utilization of the advantages of lithiophilic sites [102]. A work by the Wang group highlighted the advantages of insertion‐type TiO_2_ sites in anti‐aggregation [31]. The low lattice strain and expansion of TiO_2_ sites during Li insertion offer an advantage in firmly anchoring TiO_2_ sites within hollow carbon spheres. In this case, the long‐term dispersion of lithiophilic sites is also ensured, which is the key advantage of TiO_2_ sites (Figure 8a). The above essentials have been confirmed through in situ TEM observations. Comparing in situ TEM and HAADF‐STEM images of Ag NPs decorated hollow porous carbon spheres (Ag@N‐HPCSs) (Figure 8b–d) and TiO_2_ seeds decorated hollow porous carbon spheres (TiO_2_@N‐HPCSs) (Figure 8e,f) before and after cycling, it can be observed that TiO_2_ seeds exhibited better anti‐agglomeration property than Ag NPs on the carbon shell. After Li^+^ intercalation, HRTEM images indicated only a 4% lattice spacing expansion for TiO_2_ sites. The fast Fourier transform (FFT) patterns suggest a phase transition from the (101) plane of anatase TiO_2_ to the (011) plane of Li_0.5_TiO_2_ (Figure 8e,f), thus proving the insertion‐type mechanism of TiO_2_. Symmetric cell with TiO_2_@N‐HPCSs exhibited superior cycling stability than that of Ag@N‐HPCSs, which may be attributed to their insertion mechanism and characteristics of low‐strain and anti‐aggregation (Figure 8g).

The low expansion rate also allows TiO_2_ to serve as the primary matrix in a hollow Li host. Lu et al. integrated insertion‐type TiO_2_ nanotubes with alloying‐type Ag sites, synthesized TiO_2_ nanotube arrays decorated with Ag NPs on both edges and inner walls on Ti foil (TNT‐Ag) through a simple two‐step cathodic reduction method followed by a heat treatment process [103]. The presence of Ag NPs effectively promotes the deposition of Li inside the nanotubes, preventing Li deposition on the top of the nanotubes and inhibiting Li dendrite growth. During Li deposition process, the outer diameter of the nanotube remained almost constant while the inner radius decreased, indicating selective Li deposition inside the nanotubes without significant volume expansion of TiO_2_ and dendrite formation. This example effectively demonstrates the advantage of using TiO_2_ as the main component of hollow Li host in inhibiting volume expansion during Li deposition.

Furthermore, oxygen vacancies can be introduced into TiO_2_ by some special treatments, such as annealing in an inert atmosphere (such as Ar) [104] or vacuum [105]. The presence of oxygen vacancies has been demonstrated to alter the properties of original TiO_2_ in electrocatalysis and photocatalysis [104, 106, 107]. In view of this, Wang and colleagues constructed hollow TiO_2_ nanotubes on Cu mesh (Cu/TiO_2_), subsequently annealed in Ar to produce Cu‐loaded TiO_2_ nanotubes rich in oxygen vacancies (Cu@TNT) [108]. For comparison, Cu‐loaded TiO_2_ nanotubes with lower oxygen vacancy content (Cu@TNT‐O) were obtained by annealing the Cu/TiO_2_ in air. The introduced oxygen vacancies serve as favorable binding sites for Li^+^ adsorption, reduce Li nucleation overpotential, and enhance charge transfer capability. As expected, the Cu@TNT exhibited lower Li nucleation overpotential compared to that of Cu mesh electrode. Moreover, the voltage polarization and cycling stability of Cu@TNT@Li at different current densities outperformed those of Cu@TNT‐O@Li. The above results highlight the positive role of abundant oxygen vacancies in TiO_2_ in facilitating Li deposition. The results of XPS analysis with Ar etching further indicate that the SEI layer on the cycled Cu@TNT contained higher levels of LiF, Li_2_S, and Li_3_N, compared to that of cycled Cu mesh. These inorganic species originated from Li bis (trifluoromethanesulfonyl) imide (LiTFSI) decomposition, enhanced the mechanical stability of the SEI layer, prevented SEI layer rupture, and suppressed dendrite growth and side reactions between metallic Li and electrolyte.

Drawing from the instances illustrated, hollow Li hosts with insertion‐type metal compounds (such as TiO_2_) demonstrate minimal lattice and volume expansion during cycling, possess prolonged structural stability compared with lithiophilic metal and conversion‐type metal compounds. Nonetheless, Li hosts integrating insertion‐type metal compounds with hollow nanostructures have been less explored till now.

## 5 Conclusion and Perspectives

LMBs have garnered significant attention as promising next‐generation electrochemical energy storage devices owing to their high energy density. However, the host‐less property of metallic Li in LMAs leads to poor cycling stability. To address this, hollow Li hosts featuring lithiophilic metal or metal compound sites have passed the proof‐of‐concept stage, as they post unique nano‐structures and exhibit outstanding lithiophilicity for guiding Li deposition.

This review begins by elucidating the deposition behavior of Li in diverse hollow nanostructures, highlighting the favorable guiding effect of hollow nanosturctures on Li deposition. Furthermore, intrinsic lithiophilicity arising from interactions between metal or metal compound sites and Li is summarized. Finally, some representative instances of hollow Li hosts with metals or metal compounds are presented, covering different types of lithiophilic sites, including noble metals, non‐noble metals, conversion‐type metal compounds, and insertion‐type metal compounds. The discussed nanostructures include simple monomer hollow NPs and hollow nanofibers. The review elucidates the changes in lithiophilic sites during the cycling process and demonstrates the electrochemical performance of hollow Li hosts. Advances of the CE stability of some representative hollow Li hosts with lithiophilic metal or/and metal compound sites reported in recent years are summarized and compared in Table 1.

**TABLE 1:** Summary of recent advances in CE stability of hollow‐structured Li hosts featuring lithiophilic metal/metal compound sites.

Classification of lithiophilic site (s)	Li host	Key Lithiophilic Site (s)	Test conditions	Electrolyte	Cycle number	Refs.
Metal	Ag@CMFs	Ag	1 mA cm^−2^, 1 mAh cm^−2^	1 M LiTFSI in DOL/DME (v:v = 1:1) with 2 wt.% LiNO_3_	800	[17]
	CC‐Zn‐CMFs	Zn	1 mA cm^−2^, 1 mAh cm^−2^	1 M LiTFSI in DOL/DME (v:v = 1:1) with 2 wt.% LiNO_3_	800	[90]
	Carbon nanocapsules with Au NPs	Au	0.5 mA cm^−2^, 1 mAh cm^−2^	1.0 M LiPF_6_ in EC/DEC (1:1), with 1% VC and 10% FEC	300	[27]
	WGC	Au	1 mA cm^−2^, 1 mAh cm^−2^	10 M LiFSI in DMC	140	[28]
	MXene@Ag	Ag	1 mA cm^−2^, 1 mAh cm^−2^	1 M LiTFSI in DOL/DME (v:v = 1:1) with 1 wt.% LiNO_3_	120	[109]
	Sn@B/N/F‐CMFs	Sn	1 mA cm^−2^, 1 mAh cm^−2^	1 M LiPF_6_ in FEC/FEMC (w:w = 3:7)	650	[16]
	Ag@N‐HPCSs	Ag	1 mA cm^−2^, 1 mAh cm^−2^	1 M LiTFSI in DOL/DME (v:v = 1:1) with 2 wt.% LiNO_3_	323	[31]
	Au@PHCF	Au	1 mA cm^−2^, 1 mAh cm^−2^	1 M LiTFSI in DOL/DME (v:v = 1:1) with 1 wt.% LiNO_3_	372	[40]
	NCH@CF	Ni‐Co	3 mA cm^−2^, 1 mAh cm^−2^	1 M LiTFSI in DOL/DME (v:v = 1:1) with 2.5 wt.% LiNO_3_	250	[19]
	Li_4.4_Sn/SG	Li_4.4_Sn	1 mA cm^−2^, 2 mAh cm^−2^	1 M LiTFSI in DOL/DME	150	[110]
Conversion‐type Metal Compound	ZOS‐CF	ZnO, ZnS	1 mA cm^−2^, 1 mAh cm^−2^	1 M LiPF_6_ in EC/EMC/DMC, (v:v:v = 1:1:1) with 5 wt.% FEC	300	[30]
	MgF_2_ NSs@NGHSs	MgF_2_	1 mA cm^−2^, 1 mAh cm^−2^	1 M LiTFSI in DOL/DME (v:v = 1:1) with 0.2 M LiNO_3_	600	[29]
	ZnS@NPS‐C HRD	ZnS	1 mA cm^−2^, 1 mAh cm^−2^	1 M LiTFSI in DOL/DME (v:v = 1:1) with 0.2 M LiNO_3_	300	[97]
	CoF_2_@C‐HS	CoF_2_	1 mA cm^−2^, 1 mAh cm^−2^	1 M LiPF_6_ in EMC: FEC (v:v = 7:3)	280	[94]
Insertion‐type Metal Compound	TiO_2_@N‐HPCSs	TiO_2_	1 mA cm^−2^, 1 mAh cm^−2^	1 M LiTFSI in DOL/DME (v:v = 1:1) with 2 wt.% LiNO_3_	360	[31]
Hybrid	TNT‐Ag	TiO_2_, Ag	1 mA cm^−2^, 1 mAh cm^−2^	1 M LiTFSI in DOL/DME (v:v = 1:1) with 2 wt.% LiNO_3_	400	[103]

### 5.1 Materials Synthesis Efficiency

While the advancement of Li hosts for stabilizing LMAs has been studied for over a decade, much attention has been directed toward metal foams (e.g., Cu foam, Ni foam, etc.), layered structural materials (graphene [111, 112], reduced graphene oxide (rGO) [49, 113], MXenes [114, 115], 2D covalent organic frameworks (COFs) [116], etc.). Generally, research on Li hosts with hollow nanostructures remains relatively limited, partly due to the advanced and complex material synthesis processes required for precise structural control. Therefore, developing streamlined synthesis methods for lithiophilic hollow nanostructures could emerge as a significant research trend for future direction. Methods that allow simple formation of hollow‐structured materials could be pivotal in advancing research in this area.

Beyond synthesis complexity, the synthesis of hollow Li hosts often involves multiple steps, which potentially result in low yields (or reduced atom economy) of hollow Li hosts, particularly when employing a hard‐templating method. As large‐scale host production is essential for practical battery manufacturing, large‐scale synthesis of hollow Li hosts must be prioritized to meet the demands of practical electrode manufacturing.

The hollow Li hosts discussed in this review serve as a proof‐of‐concept demonstration of how hollow nanostructures and lithiophilic site engineering enhance the stability of LMAs. To scale up the production of hollow Li hosts to meet industrial manufacturing demands, synthesis strategies such as spray pyrolysis [117], spray drying [118, 119], biomass templating [120], or salt templating [121] could be utilized to improve both the synthesis efficiency and yield.

### 5.2 Manipulating SEI Layer Component by Conversion‐type Sites

Regarding lithiophilic functionality, metal NPs exhibit exceptional lithiophilicity but are limited to guiding uniform Li deposition via alloying reactions. They lack the ability to directly manage the composition of the SEI layer. In recent years, numerous studies have underscored the crucial role of SEI composition in influencing the cycling stability of LMAs. Therefore, compared to metal sites, conversion‐type metal compounds have great potential to generate Li compounds (such as LiF, Li_2_S, Li_3_N, etc.) as favorable components of SEI layer during cycling, while the produced metal may also be lithiophilic [122], positioning them as promising candidates for enhancing the electrochemical performance of hollow Li hosts, which is worthy of vigorous development. As mentioned above, XPS and lattice fringe/SAED pattern of in situ TEM can be used to infer the composition of SEI, and the recently developed cryo‐XPS technology enables more advanced preservation and testing quality of SEI samples [123]. Additionally, synchrotron XRD is a powerful technique as well for characterizing the SEI components resulting from conversion‐type lithiophilic species [124]. With the aid of these effective characterization techniques, it is anticipated that the stability of LMAs can achieve significant advancement, benefiting from the manipulated SEI component by hollow Li hosts featuring conversion‐type lithiophilic sites.

### 5.3 Insufficient Research on Insertion‐type Sites

Insertion‐type lithiophilic species, such as TiO_2_, exhibit great chemical and structural stability during cycling. However, hollow Li hosts with insertion‐type lithiophilic species are still seldom reported. Except for TiO_2_, there is a lack of reports on hollow Li hosts incorporating other insertion‐type species, such as Li_3_VO_4_, which have proven to have notable lithiophilicity and the capability to extend the cycling lifespan of LMAs [32]. Therefore, the development of hollow Li hosts with insertion‐type lithiophilic sites is still insufficient but represents a possible promising avenue for future research.

### 5.4 Li Host Loading Control

Numerous reports suggest that specific metals and metal compounds exhibit high Li storage capacities [125, 126, 127]. Therefore, when using hollow nanomaterials featuring lithiophilic metal/metal compound sites as Li hosts, attention should be paid to the loading of Li hosts on the electrode. Excessive Li host loading implies an abundance of lithiophilic sites in LMAs, which may lead to a significant amount of irreversible Li storage capacity in Li hosts, resulting in lower CE values in half cell tests, especially initial CE values. Additionally, in full cell tests, low initial CE values caused by the high Li storage capacity of the Li host will lead to Li^+^ capacity loss of batteries, which is crucial in anode‐free LMBs, as it can result in rapid battery failure. Therefore, it is essential to rationally adjust the loading of Li hosts when using hollow Li hosts featuring lithiophilic metals or metal compounds.

### 5.5 Opportunities for Solving Issues from Cathodes

Hollow Li hosts offer advantages in full cells. More specifically, their unique 3D structure contributes to the formation of a stable SEI layer, suppresses side reactions between Li and electrolyte, prevents the active Li loss and electrolyte run dry [128], which is beneficial for maintaining stable Li^+^ transport. Moreover, in Li‐S full cells, polysulfide shuttling leads to Li corrosion and battery failure [129, 130]. Rational design of hollow Li hosts can encapsulate Li within internal cavities, while functional shells with metal or metal compound sites can adsorb, block, or catalytically convert polysulfide, mitigating anode corrosion and improving cycling stability. Furthermore, hollow Li hosts also offer certain advantages in enabling stable high‐voltage LMBs. High‐voltage cathodes, such as LNMO, often suffer from the dissolution of transition metal ions (such as Mn^2+^) into the electrolyte during high‐voltage cycling. The released transition metal ions may shuttle to the anode side and result in anode Li corrosion [131]. Hollow Li hosts can store most Li metal within their cavities, while a functionalized shell with metals or metal compounds can be designed for capturing or delaying the transition metal ions' entrance, thereby alleviating corrosion. These concepts remain underexplored but might become promising future research trends for hollow Li host design.

## Conflicts of Interest

The authors declare no conflicts of interest.
